# Supplementary material for: Improving sepsis prediction in intensive care with SepsisAI: A clinical decision support system with a focus on minimizing false alarms
Source: PLOS Digit Health. 2024 Aug 12;3(8):e0000569. doi: 10.1371/journal.pdig.0000569 (PMC11318852; doi:10.1371/journal.pdig.0000569)
Supplement: S1 Text — (DOCX) [file pdig.0000569.s001.docx]

## **Supplementary Text S1**

**Utility Score:** A utility score was used to evaluate the early prediction of sepsis. For sepsis-positive patients, the classifiers that predict sepsis between twelve hours before and three hours after sepsis onset are rewarded with a maximum score of 1.0. The classifiers that do not predict sepsis or predict sepsis too early (more than twelve hours before sepsis onset) are penalized, where the maximum penalty for very early detection is a parameter (0.05), and the maximum penalty for late detection is also a parameter (-2.0). For sepsis-negative patients, the classifiers are penalized for false alarms. The maximum penalty for predicting sepsis for a sepsis-negative patient is 0.05, equivalent to the too-early prediction. The classifiers are neither rewarded nor penalized for not predicting sepsis for negative patients.
