# Supplementary material for: Improving sepsis prediction in intensive care with SepsisAI: A clinical decision support system with a focus on minimizing false alarms
Source: PLOS Digit Health. 2024 Aug 12;3(8):e0000569. doi: 10.1371/journal.pdig.0000569 (PMC11318852; doi:10.1371/journal.pdig.0000569)
Supplement: S1 Fig — (DOCX) [file pdig.0000569.s002.docx]

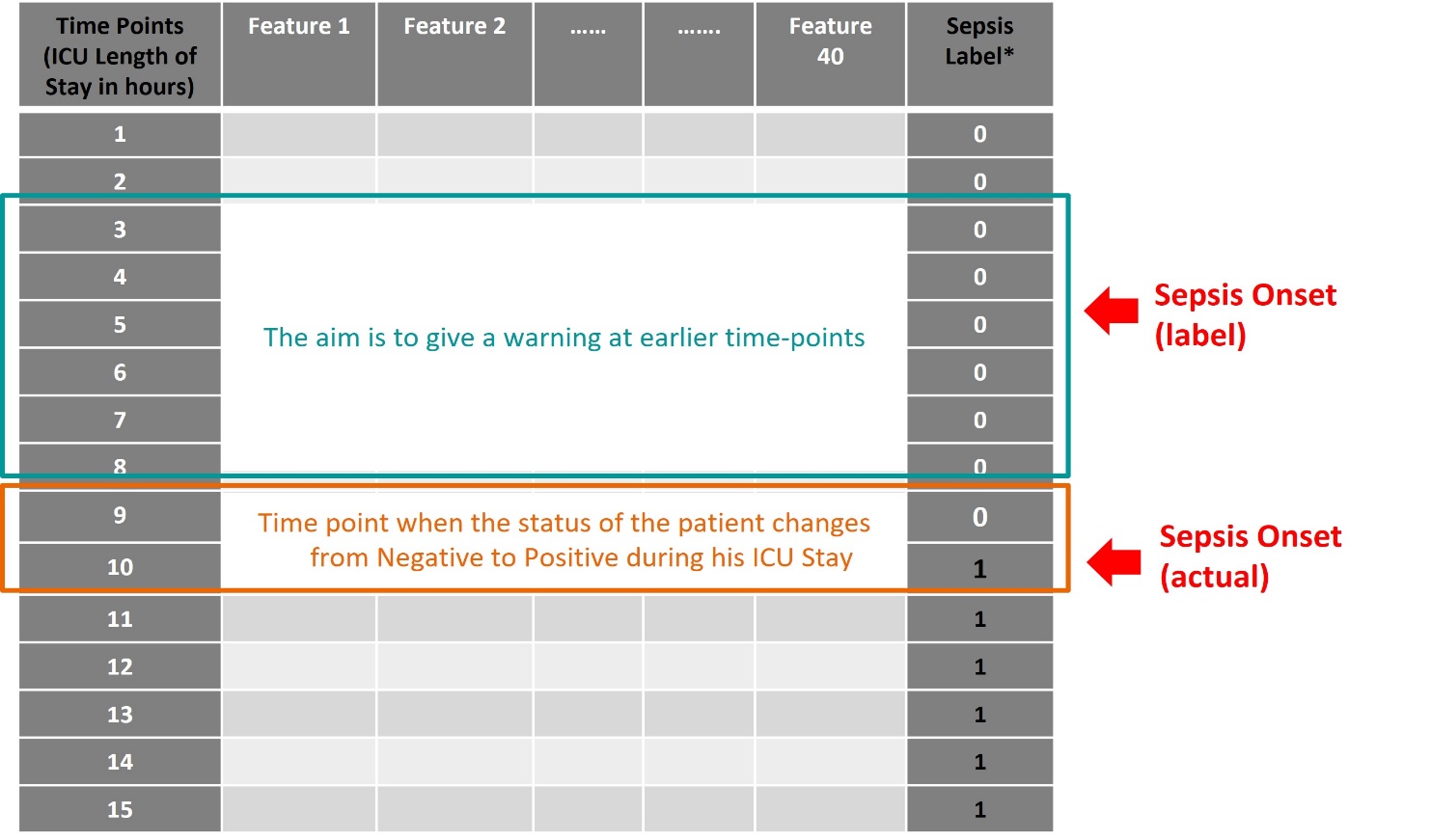


S1 Fig: The labels of sepsis onset were shifted by 6 hours from the actual onset for early prediction. For septic patients, the Sepsis label is 1 if t ≥ sepsis onset − 6 and 0 if t < sepsis onset – 6. For non-septic patients, the Sepsis label is 0.
