# Supplementary figures and images for: Improving sepsis prediction in intensive care with SepsisAI: A clinical decision support system with a focus on minimizing false alarms
Source: PLOS Digit Health. 2024 Aug 12;3(8):e0000569. doi: 10.1371/journal.pdig.0000569 (PMC11318852; doi:10.1371/journal.pdig.0000569)

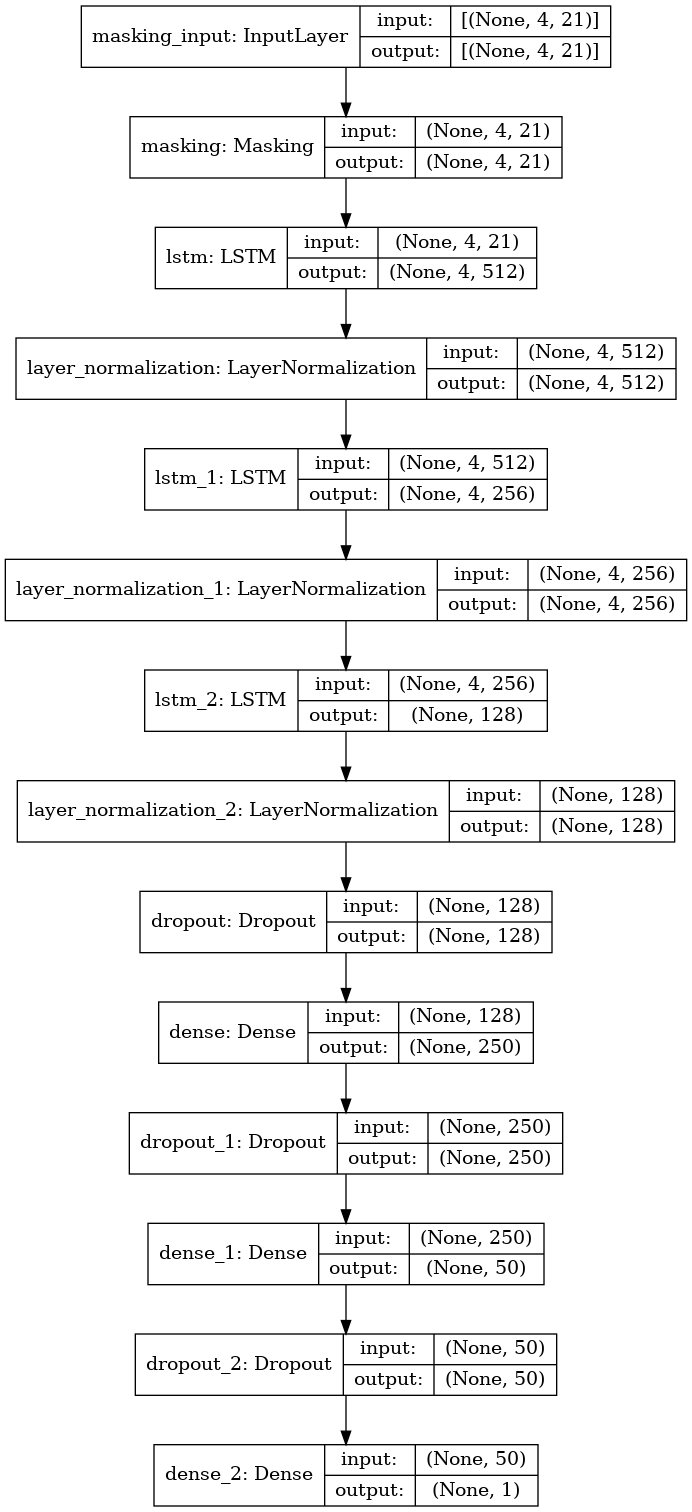


S3 Fig: Model architecture

Supplement: S3 Fig — (DOCX) [file pdig.0000569.s004.docx]

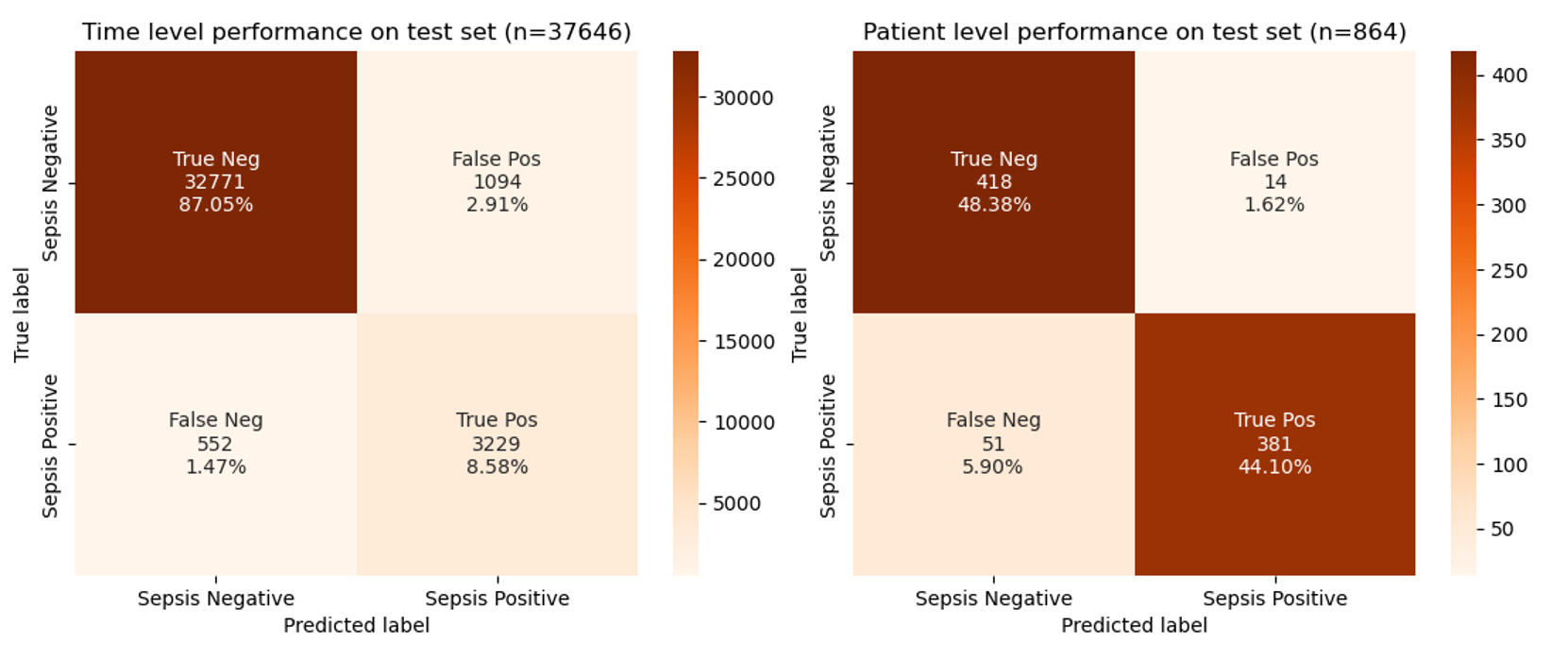


S6 Fig: Confusion matrix for alerts at the time and patient level on a balanced test set

Supplement: S6 Fig — (DOCX) [file pdig.0000569.s007.docx]

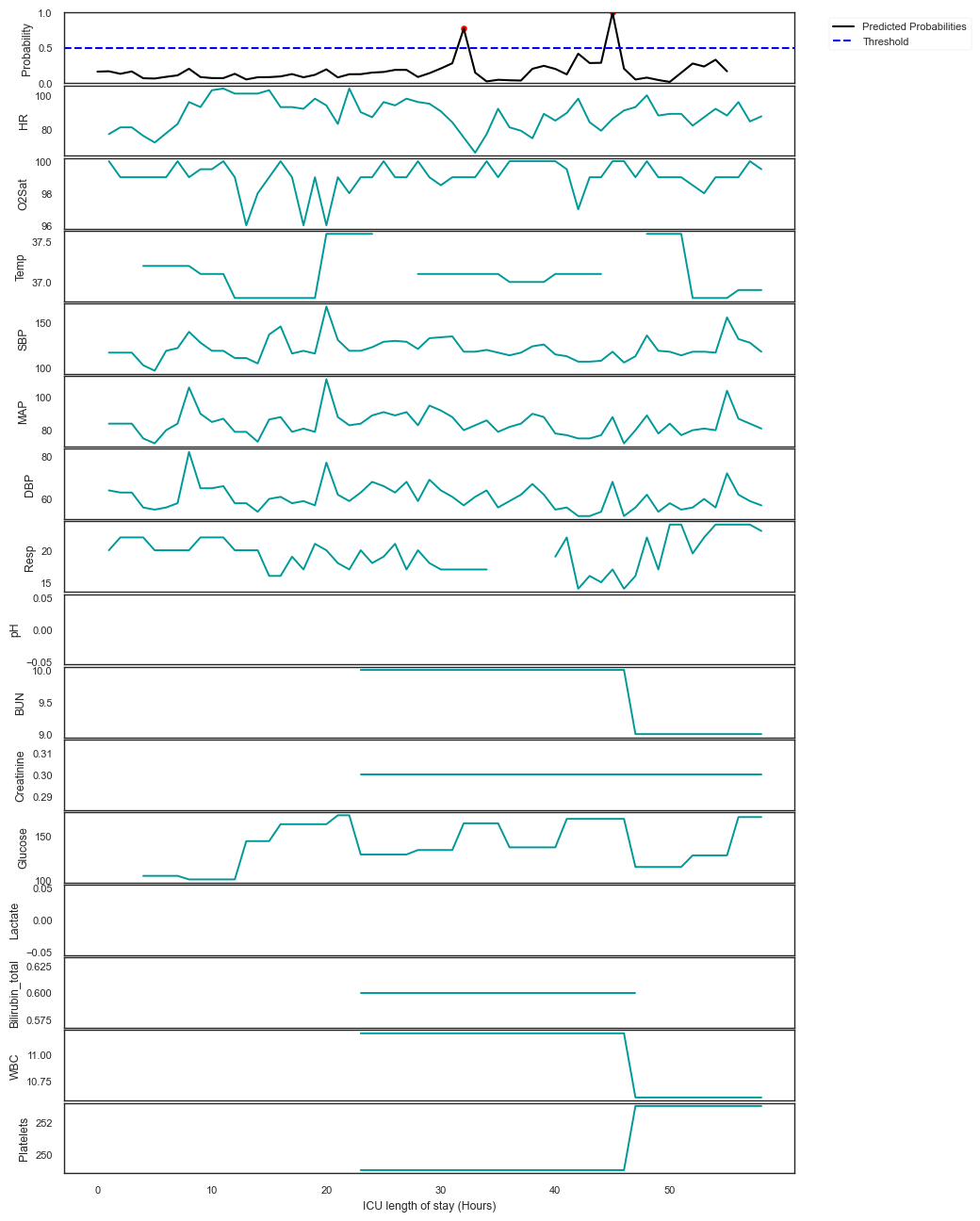


S8 Fig: Parameter Values across time

Supplement: S8 Fig — (DOCX) [file pdig.0000569.s009.docx]
