# Supplementary material for: Improving sepsis prediction in intensive care with SepsisAI: A clinical decision support system with a focus on minimizing false alarms
Source: PLOS Digit Health. 2024 Aug 12;3(8):e0000569. doi: 10.1371/journal.pdig.0000569 (PMC11318852; doi:10.1371/journal.pdig.0000569)
Supplement: S5 Fig — (DOCX) [file pdig.0000569.s006.docx]

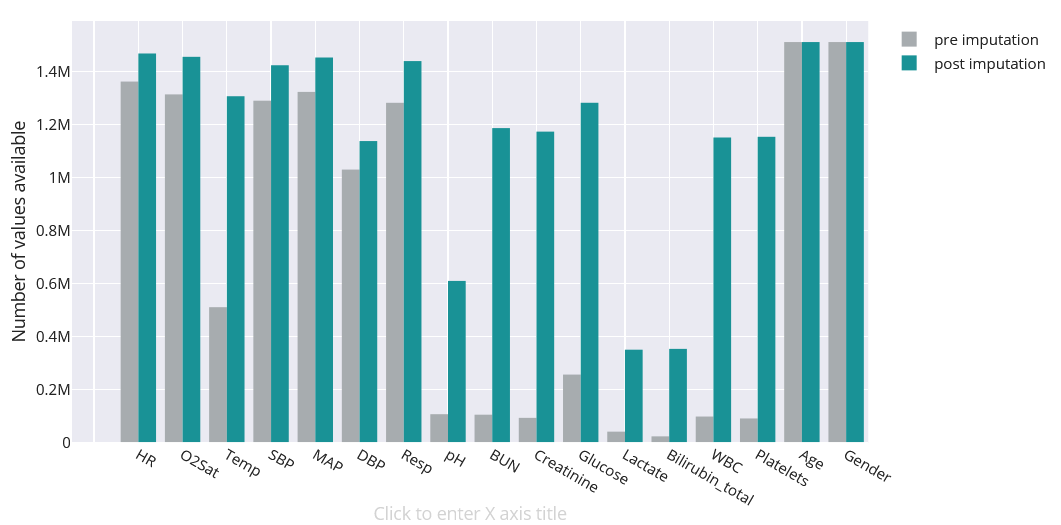


S5 Fig: Bar chart showing the parameter availability before and after imputation across selected parameters. The patients with a parameter completely absent were excluded from the analysis.
