# Supplementary material for: Improving sepsis prediction in intensive care with SepsisAI: A clinical decision support system with a focus on minimizing false alarms
Source: PLOS Digit Health. 2024 Aug 12;3(8):e0000569. doi: 10.1371/journal.pdig.0000569 (PMC11318852; doi:10.1371/journal.pdig.0000569)
Supplement: S7 Fig — (DOCX) [file pdig.0000569.s008.docx]

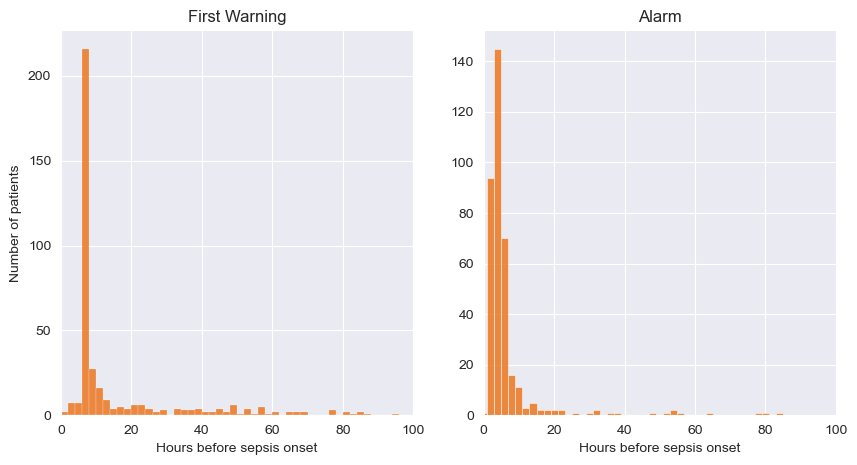


S7 Fig: Histogram depicting the number of hours before onset when the first warning was issued, and the first alert was raised
