# Supplementary material for: Improving sepsis prediction in intensive care with SepsisAI: A clinical decision support system with a focus on minimizing false alarms
Source: PLOS Digit Health. 2024 Aug 12;3(8):e0000569. doi: 10.1371/journal.pdig.0000569 (PMC11318852; doi:10.1371/journal.pdig.0000569)
Supplement: S1 Table — (DOCX) [file pdig.0000569.s010.docx]

**S2 Table**: Patient Characteristics

|  | Age | Gender |
| --- | --- | --- |
| All | 61.64 ± 16.48 | Male: 55.94% |
|  |  | Female: 44.05% |
| Train Cohort | 61.65 ± 16.47 | Male: 55.94% |
|  |  | Female: 44.06% |
| Test Cohort | 61.03 ± 16.83 | Male: 54.28% |
|  |  | Female: 45.72% |
