# Supplementary material for: Improving sepsis prediction in intensive care with SepsisAI: A clinical decision support system with a focus on minimizing false alarms
Source: PLOS Digit Health. 2024 Aug 12;3(8):e0000569. doi: 10.1371/journal.pdig.0000569 (PMC11318852; doi:10.1371/journal.pdig.0000569)
Supplement: S2 Table — (DOCX) [file pdig.0000569.s011.docx]

**S2 Table**: List of available parameters and their descriptions with units

|  | **Measurement** | **Description** |
| --- | --- | --- |
| 1 | HR | Heart rate (beats per minute) |
| 2 | 02Sat | Pulse oximetry (%) |
| 3 | Temp | Temperature (deg C) |
| 4 | SBP | Systolic BP (mm Hg) |
| 5 | MAP | Mean arterial pressure (mm Hg) |
| 6 | DBP | Diastolic BP (mm Hg) |
| 7 | Resp | Respiration rate (breaths per minute) |
| 8 | EtC02 | End tidal carbon dioxide (mm Hg) |
| 9 | BaseExcess | Excess bicarbonate (mmol/L) |
| 10 | HC03 | Bicarbonate (mmol/L) |
| 11 | Fi02 | Fraction of inspired oxygen (%) |
| 12 | pH | pH |
| 13 | PaC02 | Partial pressure of carbon dioxide from arterial blood (mm Hg) |
| 14 | Sa02 | Oxygen saturation from arterial blood (%) |
| 15 | AST | Aspartate transaminase (IU/L) |
| 16 | BUN | Blood urea nitrogen (mg/dL) |
| 17 | Alkalinephos | Alkaline phosphatase (IU/L) |
| 18 | Calcium | Calcium (mg/dL) |
| 19 | Chloride | Chloride (mmol/L) |
| 20 | Creatinine Creatinine | (mg/dL) |
| 21 | Bilirubin_direct | Direct bilirubin (mg/dL) |
| 22 | Glucose | Serum glucose (mg/dL) |
| 23 | Lactate | Lactic acid (mg/dL) |
| 24 | Magnesium | Magnesium (mmol/dL) |
| 25 | Phosphate | Phosphate (mg/dL) |
| 26 | Potassium | Potassiam (mmol/L) |
| 27 | Bilirubin. total | Total bilirubin (mg/dL) |
| 28 | TroponinI | Troponin I (ng/mL) |
| 29 | Hct | Hematocrit (%) |
| 30 | Hgb | Hemoglobin (g/dL) |
| 31 | PTT | Partial thromboplastin time (seconds) |
| 32 | WBC | Leukocyte count (count/L) |
| 33 | Fibrinogen | Fibrinogen concentration (mg/dL) |
| 34 | Platelets | Platelet count (count/mL) |
| 35 | Age | Age (years) |
| 36 | Gender | Female (0) or male (1) |
| 37 | Unit1 | Administrative identifier for ICU unit (MICU); false (0) or true (1) |
| 38 | Unit2 | Administrative identifier for ICU unit (SICU); false (0) or true (1) |
| 39 | HospAdmTime | Time between hospital and ICU admission (hours since ICU admission) |
| 40 | ICULOS | ICU length of stay (hours since ICU admission) |
| 41 | SepsisLabel | For septic patients, SepsisLabel is 1 if t tsepsis - 6 and  0 if t < tsepsis -6. For non-septic patients, SepsisLabel is 0. |
