# Supplementary material for: Improving sepsis prediction in intensive care with SepsisAI: A clinical decision support system with a focus on minimizing false alarms
Source: PLOS Digit Health. 2024 Aug 12;3(8):e0000569. doi: 10.1371/journal.pdig.0000569 (PMC11318852; doi:10.1371/journal.pdig.0000569)
Supplement: S3 Table — (DOCX) [file pdig.0000569.s012.docx]

**S3 Table**: Minimum and maximum values used for normalizing parameters.

|  | Parameters | Minimum value | Maximum value |
| --- | --- | --- | --- |
| Vital Parameters | HR | 20 | 200 |
|  | O_2_Sat | 70 | 100 |
|  | Temperature | 32 | 42.2 |
|  | SBP | 40 | 240 |
|  | MAP | 20 | 250 |
|  | DBP | 20 | 150 |
|  | Resp | 1 | 50 |
| Lab Parameters | pH | 7 | 7.7 |
|  | BUN | 1 | 120 |
|  | Creatinine | 0 | 10 |
|  | Bilirubin | 0 | 15 |
|  | WBC | 0 | 40 |
|  | Platelets | 1 | 650 |
|  | Glucose | 10 | 320 |
|  | Lactate | 0 | 12 |
| Demographic Parameter | Age | 18 | 100 |
