# Supplementary material for: Improving sepsis prediction in intensive care with SepsisAI: A clinical decision support system with a focus on minimizing false alarms
Source: PLOS Digit Health. 2024 Aug 12;3(8):e0000569. doi: 10.1371/journal.pdig.0000569 (PMC11318852; doi:10.1371/journal.pdig.0000569)
Supplement: S4 Table — (DOCX) [file pdig.0000569.s013.docx]

| Metric | Time level | Patient Level |
| --- | --- | --- |
| Accuracy | 95.62% | 92.47% |
| Specificity | 96.76% | 96.75% |
| Sensitivity | 85.40% | 88.19% |
| F1-score | 79.68% | 92.14% |
| PPV | 74.69% | 96.45% |
| NPV | 98.34% | 89.12% |

**S4 Table**: Model performance at the time and patient level considering alerts. Probability threshold = 0.5, $\omega=5$ , $x$ = 3
