# Supplementary material for: Improving sepsis prediction in intensive care with SepsisAI: A clinical decision support system with a focus on minimizing false alarms
Source: PLOS Digit Health. 2024 Aug 12;3(8):e0000569. doi: 10.1371/journal.pdig.0000569 (PMC11318852; doi:10.1371/journal.pdig.0000569)
Supplement: S5 Table — (DOCX) [file pdig.0000569.s014.docx]

**S5 Table**: Model performance (patient level) w.r.t sepsis onset from ICU admission

| Sepsis onset after ICU admission | Number of Patients | Prediction Accuracy |
| --- | --- | --- |
| 0 to 12 hours | 142 | 83.80% |
| 12 to 24 hours | 57 | 84.21% |
| 24 to 48 hours | 73 | 84.93% |
| 48 to 72 hours | 48 | 93.75% |
| 72+ hours | 112 | 87.75% |
