# Supplementary material for: Improving sepsis prediction in intensive care with SepsisAI: A clinical decision support system with a focus on minimizing false alarms
Source: PLOS Digit Health. 2024 Aug 12;3(8):e0000569. doi: 10.1371/journal.pdig.0000569 (PMC11318852; doi:10.1371/journal.pdig.0000569)
Supplement: Attachment — Submitted filename: Response to Reviewers.docx [file pdig.0000569.s015.docx]

**RESPONSE TO REVIEWERS**

**Reviewer #1: This is a moderately sized, retrospective, ICU, sepsis-3 machine learning prediction time series analysis. There is inherent value in predicting severe infection and there is theoretical potential benefit in earlier treatment and management. Strengths of this analysis include the detailed time windowing of the prediction period and the accuracy. The analysis of the trade off’s between windowing, and number of alerts is useful. Weaknesses include the limitation to an ICU cohort, that by definition are already closely monitored, and lack of specifics about the components of Sepsis-3 that are being predicted.**

We sincerely thank Dr. Edward James Schenck for the thoughtful review and constructive feedback. We are especially grateful for the recognition of the strengths of our work, which align perfectly with the key points we aimed to highlight. We also appreciate the reviewer's identification of areas for improvement. We have carefully considered the suggestions and incorporated necessary revisions in the revised manuscript. We believe these changes address the raised concerns and trust that the reviewer will find the revised version satisfactory.

**Reviewer #1:**

**1. Central to the derivation of benefit from prediction is a deep understanding of the disparate components of the current epidemiologic definition of sepsis, Sepsis-3. This definition is a combination of cultures (for diagnosis), antibiotics (treatment) and an increase or absolute SOFA score > 2 (a presumed effect of the infection). Each of these components has a different biologic and sociologic meaning. Specifically, the increase in SOFA score may be the result of a severe infection but could also be caused by mechanical (such as a pulmonary embolism or bleeding) or iatrogenic complications. Moreover, the decisions to attempt to diagnosis or treat a potential infection are reflective of the sociology of the unit under consideration. Thus, predicting sepsis under this construct is NOT PREDICTING an infection. It is predicting several things that are tangentially related to an infection. That said, the physionet challenge was to predict sepsis in this construct, so it is not the fault of the authors. For the results to be put into context, it must be clear as to what is being predicted. As these events occur in series, the final component finishes the definition. Is the prediction antibiotic ordering, a change in SOFA, or the ordering of cultures? It is most important to predict the active orders as this may lead to earlier treatment, however if the majority of cases are predicting a change in SOFA, when antibiotics are already in place then the prediction has less value.**

We appreciate the reviewer's insightful comment and detailed overview of the different components of the Sepsis definition. We fully concur that in the given construct the algorithm is not predicting an infection, however, the intent was also not to predict just infection.

Following the Sepsis-3 definition, Sepsis Challenge defines Sepsis onset (t_sepsis_) as the earlier of t_suspicion_ and t_SOFA_ (given t_SOFA_ occurs no more than 24 hours before or 12 hours after t_suspicion_) where:
t_SOFA_ is the time point of two-point deterioration in SOFA score within 24 hours

t_suspicion_ is the clinical suspicion of infection identified as the earlier timestamp of IV antibiotics administration (for at least 72 consecutive hours) and blood cultures within a specified duration.

Based on this construct, we deduce that the algorithm does not predict isolated events, such as changes in SOFA scores, culture orders, or antibiotic administration. Instead, it focuses on identifying organ failure within the context of infection. This is supported by the negative control group, which included patients meeting only the organ failure criteria or only the suspected infection criteria. The algorithm's performance indicates that it predicts sepsis only when both criteria are met: organ failure concurrent with an infection. Notably, while the suspicion of infection criteria considers factors like culture orders and antibiotic use, these actions are not directly incorporated into the algorithm's training. Instead, it continuously monitors representative markers of infection (e.g., WBC, temperature, HR) and organ function (e.g., creatinine, BUN for kidneys, bilirubin for liver) over time. We believe this approach allows the algorithm to identify the co-occurrence of infection and organ failure. We acknowledge the reviewer's valuable point regarding the need for clarity on the algorithm's predictive capabilities. To address this, we have added a section in the revised manuscript's discussion explicitly addressing this aspect (Page 19, lines 335-342).

We concur with the reviewer's observation regarding the clinical utility when antibiotics are already administered. However, it's crucial to note that the algorithm predicts sepsis four to six hours before its onset, which precedes the 't_suspicion_' window (including antibiotic administration) and t_SOFA_. This implies that when the algorithm accurately identifies septic patients, it does so before the initiation of antibiotic therapy. While analyzing the time interval between antibiotic administration and prediction would have provided valuable insights into the algorithm's true impact, we were unable to perform this analysis due to the absence of antibiotic administration time data in the Sepsis Challenge.

**2. The results pertaining to the look ahead period, the warning v alert accuracy etc need to be referenced to the above concern. If the insights are to be actionable, then the components predicted must be clear.**

We acknowledge the reviewer's valid point regarding the need for actionable insights from the algorithm's predictions. As elaborated in the previous response, the algorithm prioritizes identifying the potential for organ failure within the context of an infection. We believe the additional text included in the revised manuscript's discussion section effectively clarifies this for readers and contextualizes the algorithm's predictive window.

**3. The selected derived features include the outcome. More specifically, the pSOFA is part of the outcome definition of sepsis 3. Please justify this selection.**

We thank the reviewer for raising the concern regarding the possible circularity (outcome as input parameters) in our approach. To clarify, we did not use the complete SOFA score, which served as one of the parameters for ground truth for labeling patients in the Sepsis Challenge, as an input feature. Instead, we employed a partial SOFA score (pSOFA) which only takes few parameters into consideration (mainly, Platelets and Bilirubin). Sepsis challenge did not provide all the information required to calculate the complete SOFA score. Specifically, the following essential parameters were missing and hence did not contribute to SOFA Score:

- GCS score for CNS,
- administration of dopamine and dobutamine for Cardiovascular,
- urine output measurement for Renal, and
- PaO_2_ and FiO_2_ (available in the dataset but missing for over 50% of sepsis positive patients) for respiration.

Thus, the resulting pSOFA score is an adaptation of SOFA score tailored to the information provided by the Sepsis Challenge dataset. This allows the us to incorporate some clinical domain knowledge into our inputs without potentially having circularity in our algorithm. We have added this information in the revised version of the manuscript (Page 8, lines 150-154) to avoid confusion.

**4. The comparator scoring systems are not designed to predict in ICU sepsis. SIRS, and NEWS were designed as screening concepts for patients outside of the ICU. The SOFA score is not a prediction system, it is multiple organ dysfunction score and part of the definition of sepsis, without the concern for infection. This speaks to the relatively selected population for this exercise, see comment 6 below.**

We acknowledge that the comparator scoring systems, such as SIRS and NEWS, were originally designed as screening tools for patients outside of the ICU, and the SOFA score is primarily used to assess multiple organ dysfunction rather than predict sepsis in isolation. We recognize that the use of these scoring systems may not fully capture the complexity of sepsis detection in the ICU setting. However, given the absence of standardized criteria for sepsis detection and the lack of implementable comparable research, we chose to utilize these commonly employed scoring systems as comparators in our study. The existing algorithms which are cited in the introduction section of the manuscript cannot be implemented due to difference in training and testing data, variability in the feature set, unavailability of model or complete implementation details. We have now mentioned this as the limitation of the study under the discussions section of the revised manuscript (Page 21, lines 379-383).

**5. It may be useful to contextualize the results of this exercise in terms of the eventual outcomes of the predicted sepsis cases v those without the event. Specifically, what are the outcomes for those with predicted sepsis v without predicted sepsis. How many had a documented infection? How many continued antibiotics for more than the initial dose? How many survived the ICU stay?**

We thank the reviewer for their keen interest and share their curiosity regarding documented infections, antibiotic use duration, and patient survival outcomes during ICU stay. Unfortunately, the Sepsis Challenge dataset, the foundation of this study, lacks information on these specific aspects for patients. While we acknowledge the significance of understanding these outcomes in relation to our model's predictions, we are constrained by the limitations of the available data.

It's important to emphasize that despite these limitations, the Sepsis Challenge dataset remains one of the most well-structured and curated publicly available resources for sepsis prediction research. We are glad to inform the reviewer that our ongoing research actively investigates these very aspects they've highlighted. We're hopeful to provide additional insights and explore the algorithm's impact in future publications.

**6. The framing of the manuscript speaks of general sepsis prediction; however this is a selected ICU cohort. Sepsis is one of the most common causes of admission to an ICU, so selecting ICU onset sepsis (meaning not present on admission) creates bias that may limit the generalizability of any findings. This needs to be discussed and put into context. Moreover, why was the 4 hour cut off selected for inclusion into the cohort? Sepsis 3 onset 6 hours after arrival to the ICU is much different than Sepsis onset on day 3 after arrival to the ICU. It may be argued that the 6 hour sepsis onset is simply a labeling problem, meaning that everyone knew that the patient had sepsis prior to criteria being met. The day 3 sepsis is potentially a new event.**

We appreciate reviewer’s feedback and would like to address these points accordingly. Firstly, we agree that the framing of our manuscript as general sepsis prediction may not fully reflect the specific focus on predicting hospital-acquired sepsis in the ICU setting. We have revised the manuscript accordingly to clarify this point, as highlighted in (Page 1 – line 17, Page 3 - line 46, and Page 5 - line 101).

Regarding the selection criteria of patients with sepsis onset greater than 4 hours after ICU admission, we acknowledge the reviewer's point. We would like to highlight that this filtering was pre-applied by the Sepsis Challenge dataset creators, likely to ensure sufficient data points before sepsis onset for shifting labels and enabling early prediction.

We acknowledge the clinical distinction between patients getting sepsis shortly after ICU admission and those with onset occurring after a few days. However, our analysis demonstrates consistent algorithm performance across both scenarios, as outlined in our response to the 8th question. We agree that in the first scenario, the patients might already be suspected of having sepsis, potentially reducing the immediate predictive utility of the algorithm. This detection capability, even if not strictly "early prediction," could potentially still be beneficial.

**7. Figure 3 has some value but lacks clinical context. It would be useful to display the collected set of features, e.g. vital signs, antibiotic orders, and lab values etc for the referent patients in the panels.**

The initial representation of the Figure was designed to reflect our envisioned interface at the time of deployment, where showing all features might have overwhelmed users with too much information. However, we recognize the value of providing a more detailed depiction of the collected features for better clinical context. We have updated the figure to include the lab and vital parameters (Supplementary Figure S8). Unfortunately, we do not have antibiotic order information and hence cannot be added into the figure.

**8. When were the sepsis events in this study relative to ICU admission? Was performance different in different epochs after ICU admission?**

Thank you for the insightful question. Below is the data detailing the requested analysis. The findings indicate consistent performance regardless of the timing of sepsis occurrence relative to ICU admission. We have added this information in the supplementary section (Supplementary Table S5) which is referred in the main manuscript in Page 15, lines 274-279.

| **Sepsis Onset after ICU admission** | **Number of Patients** | **Accuracy** |
| --- | --- | --- |
| 0 to 12 hours | 142 | 83.80% |
| 12 to 24 hours | 57 | 84.21% |
| 24 to 48 hours | 73 | 84.93% |
| 48 to 72 hours | 48 | 93.75% |
| 72+ hours | 112 | 87.75% |

**9. What was the rationale to rescale with the utilized formula, line 139?**

Thank you for your inquiry. The rationale behind rescaling the data using the formula at line 139 was to amplify even subtle changes in the parameter values. By rescaling the data, we aimed to enhance the model's sensitivity to small fluctuations in vital signs and laboratory parameters, which are often indicative of early signs of deterioration in critically ill patients. This rescaling strategy was empirically derived to ensure that the model can effectively learn and respond to subtle changes in patient status, thereby improving its overall performance.

**Reviewer #2: Thank you for the opportunity to review the manuscript titled “Improving sepsis prediction in intensive care with SepsisAI: a clinical decision support system with a focus on minimizing false alarms”. This paper presents a commendable effort in developing a machine learning algorithm to predict the onset of sepsis in ICU patients. The methodology involving a detailed evaluation of the model at both the "time level" and "patient level" is particularly noteworthy. The approach demonstrates a lower false alarm rate as compared to other scoring systems. However, I have a few specific comments and suggestion that could potentially enhance the quality and clarity of the research:**

We sincerely thank the reviewer for their positive feedback on our study. It is truly motivating and serves as a valuable encouragement for our work. We appreciate the constructive suggestions provided, and we have incorporated most of them into the revised manuscript. We believe these revisions have enhanced the clarity and overall quality of the manuscript.

**1)	The paper would benefit from a more detailed description of the patient population demographics in the dataset. Understanding the age, gender, race, and other demographic factors is crucial for assessing the generalizability of the model.**

We thank the reviewer for the feedback. We agree that providing a detailed description of the patient population demographics is important for assessing the generalizability of our model. We have included the available demographic information, namely age, and gender, in the supplementary materials (Supplementary Table 1). While we recognize the importance of additional demographic factors such as race, we unfortunately do not have access to this data in our dataset.

**2)	The method used for splitting the data into training, validation, and test sets needs further clarification. It's unclear whether the data was randomly split or if there was another stratification process involved.**

We thank the reviewer for bringing this to our attention. We have revised the manuscript to clarify the data splitting process. Specifically, the data was randomly split at the patient level. This information is now included in the manuscript on Page 6, lines 117-118.

**3)	Regarding the second held-out test set, the number of positive cases (34) seems too small for a comprehensive evaluation of the model's performance. If feasible, expanding this to include 432 positive cases and 6000 negative cases could provide a more rigorous assessment of the model's predictive capabilities.**

We appreciate reviewer’s thorough consideration of our model evaluation process. We would like to provide additional context to clarify our approach. As mentioned in lines 160 to 164 (Page 8) of our manuscript, we undertook an under-sampling approach for the negative samples, drawing from all available non-septic patients excluding the held-out test set. This method was implemented at the matrix level to ensure maximal coverage of non-septic patients and to introduce sufficient heterogeneity into our training cohort. Consequently, we were left with 432 negative patients that remained unseen by the model during training. Maintaining a prevalence ratio of 7.2% in our test set necessitates the inclusion of 34 positive cases. Given the constraints imposed by the limited availability of negative cases within the held-out test set, expanding the prevalence-based test set to include 432 positive cases and 6000 negative cases is unfortunately not feasible.

**4)	While the forward-filling imputation strategy to address missing values is a smart choice, there's room to reconsider the time frame for certain rapidly changing laboratory values in sepsis conditions, such as lactate, some CBC parameters, PT, or procalcitonin. Employing a 12-hour window instead of a 24-hour one might yield more accurate results, given that laboratory values can fluctuate significantly in a short period for ICU patients.**

We do acknowledge the potential benefits of employing a narrower time frame, such as a 12-hour window, for rapidly changing laboratory values in sepsis conditions. However, careful analysis of our dataset reveals that most lab values are typically reported at a 24-hour frequency (Supplementary Figure S4). In this case, implementing a 12-hour forward-filling strategy would introduce a significant amount of missing data, potentially compromising the integrity of our data and subsequent analysis. It is to be noted that for parameters that are available more frequently, the latest values would be updated accordingly. Nevertheless, a common and liberal threshold was selected for consistency across parameters, ensuring that we don’t overfit a particular dataset or hospital. We believe that maintaining a 24-hour window strikes the best balance between data completeness and capturing relevant clinical information.

**5)	Evaluating the model in a prospective patient population would be helpful to understand its real-world utility. I am interested in knowing the next steps. Maybe the authors can discuss about it.**

We greatly appreciate the reviewer's suggestion and curiosity. We concur that evaluating our model in a prospective patient population is crucial for comprehensively assessing its real-world utility. Our ongoing efforts are dedicated to precisely this endeavor, as we seek to validate our approach further and deploy it prospectively within hospital setting. Additionally, we are actively investigating the potential impact of our algorithm on length of stay and mortality reduction. We are eager to share our progress and findings on these fronts in future publications. We have taken note and updated the discussion section - where we discussed the future work (Page 21, lines: 384-388)**.**
