# Supplementary material for: Improving sepsis prediction in intensive care with SepsisAI: A clinical decision support system with a focus on minimizing false alarms
Source: PLOS Digit Health. 2024 Aug 12;3(8):e0000569. doi: 10.1371/journal.pdig.0000569 (PMC11318852; doi:10.1371/journal.pdig.0000569)
Supplement: Attachment — Submitted filename: Response to Reviewers.docx [file pdig.0000569.s016.docx]

**RESPONSE TO REVIEWERS**

**Reviewer #1:**

1. **It is still not clear to me whether the sepsis challenge allows for the prediction of T suspicion of sepsis and T sofa and what these mean. The authors should strongly consider a sensi􀆟vity analysis to determine whether Tsuspicion can be predicted (this is an ac􀆟on by clinicians cultures and an􀆟bio􀆟cs) and may be more valuable. “Sepsis” onset may not be valuable without knowing the components. I understand that this may be limited by the available data of the sepsis data challenge. This is not the fault of the authors but the fault of the challenge organizers.**

We thank the reviewer for the comments and appreciate the perspective that predicting Tsuspicion might be more valuable. We would have been pleased to present the sensitivity analysis for Tsuspicion; unfortunately, the Sepsis Challenge dataset does not provide the specific time-points for Tsuspicion and Tsofa, making it impossible for us to perform a detailed sensitivity analysis for Tsuspicion. We are grateful that the reviewer acknowledges that this is a limitation of the dataset and beyond our control. Nonetheless, we present here an explanation of how the requested aspect might already be covered in our analysis and this limitation does not impact the outcome:

According to the definition used, the time-point of sepsis onset is identified as the earlier of two events: suspicion of infection (indicated by culture and antibiotic orders) and organ dysfunction (indicated by a change in the SOFA score). This leads to two possible scenarios:

Scenario 1: If the suspicion of infection occurs first, the time-point of sepsis onset is the same as the time-point of suspicion of infection. In such cases, the algorithm is indeed predicting suspicion of infection.

Scenario 2: If organ dysfunction occurs first, the time-point of organ dysfunction is considered the time-point of sepsis onset, provided that suspicion of infection follows within 24 hours. Which means that in such cases, when the algorithm raises an alarm, it is happening well before the suspicion of infection.

Therefore, in both scenarios, the algorithm is raising an alarm before the event of suspicion of infection. Consequently, performing a sensitivity analysis of Tsuspicion would yield the same performance results. Moreover, in Scenario 2, the lookahead time will likely improve by several hours.

We also want to highlight that apart from the limitation of not providing the supportive information of tagging the patients with sepsis onset, it is still a widely used dataset by sepsis researchers [1-5] as it labels data according to Sepsis-3 clinical criteria [6-8]. Many past and current studies on sepsis algorithms, which used datasets other than Sepsis Challenge, also employ the same construct for tagging and predicting sepsis onset in their datasets [9-12], suggesting its wide acceptance.

We hope that the reviewer will find the explanation satisfactory.

References:

1. Shashikumar, Supreeth P., et al. "Artificial intelligence sepsis prediction algorithm learns to say “I don’t know”." *NPJ digital medicine* 4.1 (2021): 134.
2. Moor, Michael, et al. "Early prediction of sepsis in the ICU using machine learning: a systematic review." Frontiers in medicine 8 (2021): 607952.
3. Yang, Meicheng, et al. "An explainable artificial intelligence predictor for early detection of sepsis." Critical care medicine 48.11 (2020): e1091-e1096.
4. Li, Xiang, et al. "A time-phased machine learning model for real-time prediction of sepsis in critical care." Critical Care Medicine 48.10 (2020): e884-e888.
5. Zhao, Xin, Wenqian Shen, and Guanjun Wang. "Early prediction of sepsis based on machine learning algorithm." Computational Intelligence and Neuroscience 2021.1 (2021): 6522633.
6. Seymour, Christopher W., et al. "Assessment of clinical criteria for sepsis: for the Third International Consensus Definitions for Sepsis and Septic Shock (Sepsis-3)." *Jama* 315.8 (2016): 762-774.
7. Singer, Mervyn, et al. "The third international consensus definitions for sepsis and septic shock (Sepsis-3)." *Jama* 315.8 (2016): 801-810.
8. Shankar-Hari, Manu, et al. "Developing a new definition and assessing new clinical criteria for septic shock: for the Third International Consensus Definitions for Sepsis and Septic Shock (Sepsis-3)." *Jama* 315.8 (2016): 775-787
9. Persson, Inger, et al. "A machine learning sepsis prediction algorithm for intended intensive care unit use (NAVOY Sepsis): proof-of-concept study." *JMIR formative research* 5.9 (2021): e28000.
10. Nemati, Shamim, et al. "An interpretable machine learning model for accurate prediction of sepsis in the ICU." *Critical care medicine* 46.4 (2018): 547-553.
11. Wardi, Gabriel, et al. "Predicting progression to septic shock in the emergency department using an externally generalizable machine-learning algorithm." *Annals of emergency medicine* 77.4 (2021): 395-406.
12. Olabisi, Olayemi, Mohamed Bader-El-Den, and James McNicholas. "Timely Sepsis Prediction in ICU Patients Using Baised Ensemble Machine Learning Approach." *Available at SSRN 4427530*.
2. **pSOFA is adding circularity to the predic􀆟on model. Either remove it from the model or discuss it as a limita􀆟on.**

Regarding the circularity concerns from pSOFA, we have discussed this as a limitation in the revised version of the manuscript (line number 383-384).

**Reviewer #2: Thank you for addressing my questions. I think the manuscript is acceptable for publication.**

We sincerely thank the reviewer for finding our responses satisfactory and for accepting the manuscript for publication.
